# Non alcoholic fatty liver disease in a young male with celiac disease

**DOI:** 10.11604/pamj.2019.32.25.16345

**Published:** 2019-01-16

**Authors:** Zain Majid, Ghous Bux Somoro, Muhammad Manzoor Ul Haque, Raja Taha Yaseen, Shoaib Ahmed Khan, Inamullah Khan Achakzai, Muhammad Ali Khalid, Nasir Hassan Luck

**Affiliations:** 1 Department of Hepatogastroenterology, Sindh Institute of Urology and Transplantation (SIUT), Karachi, Pakistan

**Keywords:** Celiac disease, NASH, gluten free diet, BMI

## Abstract

A young emaciated male, known case of celiac disease came with complaints of diarrhea along with 5kgs of weight loss in 3 months' time. He had severe electrolyte abnormalities along with low albumin, low calcium and a high phosphate with deranged liver function test. Ultrasound abdomen had shown fatty liver. Nutrition consult was sought and he was found to have a BMI of 6.8kg/m^2^. He was started on nutrition support along with supportive therapy, which resulted in weight gain and improvement in his condition.

## Introduction

Celiac disease is an autoimmune disease characterized by gluten sensitivity, affecting genetically affected individuals [1]. It has intestinal and many extra intestinal features, while diagnosis requires both serological evidence along with small intestinal biopsy suggestive of the disease [1].

## Patient and observation

**Figure 1:**
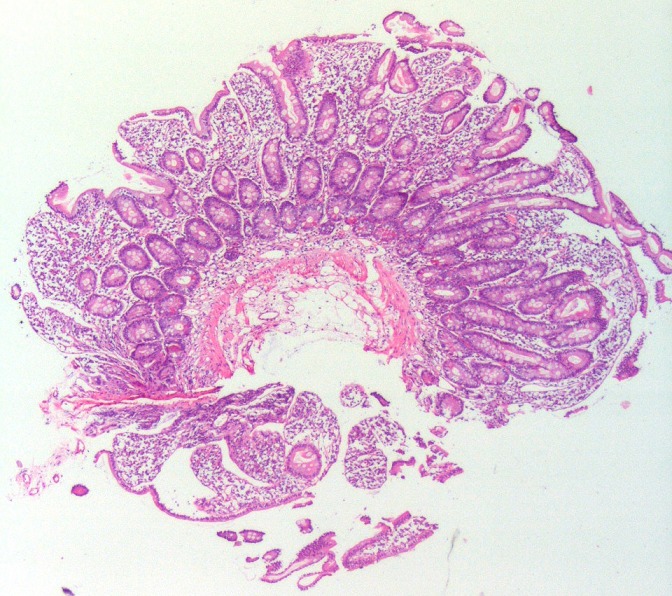
Low-power view showing moderate villous stunting and crypt hyperplasia, consistent with Marsh class 3b (H&Ex40)

**Figure 2:**
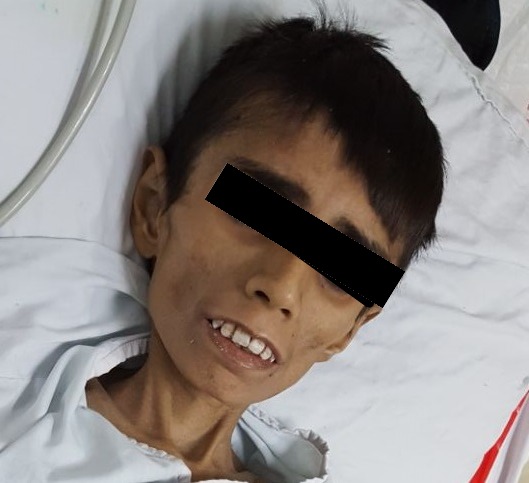
Physical appearance of the patient

A 22-year-old emaciated male presented to the gastroenterology clinic of our department with complaints of diarrhea and weight loss since the last 3 months. His old record revealed him to be a diagnosed case of celiac disease, having been diagnosed 3 years prior to this admission, which was based upon his Tissue Transglutaminase serology (TTG serology) and duodenal biopsy report (Figure 1). He was later started on gluten free diet (GFD) and was initially compliant on it for a year. Later on, he became non-compliant and had been so ever since then. Currently his loose stools were watery in consistency, occurring 7-8 times per day. They were foul smelling, being difficult to flush, associated with tenesmus and with weight loss of around 5 kgs during this same time period. He did not complain of decrease in his appetite during this time nor had any history of fever, nausea, vomiting or of dysphagia. On examination, he appeared severely wasted with thin brittle hair along with dry skin and had prominent costal margins (Figure 2). His initial lab reports showed a low hemoglobin 10.2 g/dL along with a low platelet count 102,000 10^9^fL. He was also found to have a severe electrolyte in-balance along with a low potassium (2.5 mEq/L), a low calcium (7.6 mg/dL), low albumin 1.8 g/dL, high phosphate 6.4 mg/dL and deranged LFTs with a TBR 0.48 U/L, ALP 303 U/L, SGPT 101 U/L, SGOT 65 U/L GGT 29 U/L. His iron profile, B12 and folate levels were sent along with lipid profile, which were all within normal limits. Ultrasound abdomen was later done and revealed diffuse fatty liver. His currently height was 132cm, while his weight was of 11.5kg along with a BMI 6.8 kg/m^2^. A nutritionist was immediately taken on board and he was started on blendized diet along with total parenteral nutrition and with replacement of his deranged electrolytes. His condition later improved within a week followed by weight gain of 3 kgs in one week. His oral intake gradually improved and his intravenous nutritional support was gradually tapered off. He regained 4 kg of weight and was later discussed and advised for regular follow-ups.

## Discussion

Many forms of hepatic diseases can be seen in those affected with celiac disease, including non alcoholic fatty liver disease and Nonalcoholic steatohepatitis (NASH) [2]. Non-alcoholic fatty liver disease or NAFLD is said to the commonest cause of chronic liver disease in the developed world [3, 4]. Celiac disease is seen in 4 to 13% in those having NASH [2]. The exact mechanism of this association is not known but it is plausible that this may be due to the increased intestinal permeability along with malabsorption of choline, a lipotropic factor and vitamin B6 [2]. According to one study, celiac disease is more often seen amongst those NAFLD patients having a BMI less than 27 kg/m^2^ [5]. This risk of NAFLD in celiac disease is mainly seen during the first year of life and does remain even after that [6]. It is said that GFD may help those celiac patients who have NAFLD, but the long term effects of this are still unknown [7].

## Conclusion

Our case report suggests the importance of timely checking of LFTs in celiac disease patients, which could help prevent further complications in these patients.

## Competing interests

The authors declare no competing interests.
